# Extremely stretchable thermosensitive hydrogels by introducing slide-ring polyrotaxane cross-linkers and ionic groups into the polymer network

**DOI:** 10.1038/ncomms6124

**Published:** 2014-10-08

**Authors:** Abu Bin Imran, Kenta Esaki, Hiroaki Gotoh, Takahiro Seki, Kohzo Ito, Yasuhiro Sakai, Yukikazu Takeoka

**Affiliations:** 1 Department of Molecular Design and Engineering, Graduate School of Engineering, Nagoya University, Furo-cho, Chikusa-ku, Nagoya 464-8603, Japan; 2 Department of Advanced Materials Science, Graduate School of Frontier Sciences, The University of Tokyo, 5-1-5 Kashiwanoha, Kashiwa, Chiba 277-8561, Japan

## Abstract

Stimuli-sensitive hydrogels changing their volumes and shapes in response to various stimulations have potential applications in multiple fields. However, these hydrogels have not yet been commercialized due to some problems that need to be overcome. One of the most significant problems is that conventional stimuli-sensitive hydrogels are usually brittle. Here we prepare extremely stretchable thermosensitive hydrogels with good toughness by using polyrotaxane derivatives composed of α-cyclodextrin and polyethylene glycol as cross-linkers and introducing ionic groups into the polymer network. The ionic groups help the polyrotaxane cross-linkers to become well extended in the polymer network. The resulting hydrogels are surprisingly stretchable and tough because the cross-linked α-cyclodextrin molecules can move along the polyethylene glycol chains. In addition, the polyrotaxane cross-linkers can be used with a variety of vinyl monomers; the mechanical properties of the wide variety of polymer gels can be improved by using these cross-linkers.

## Abstract (web summary)

Stimuli-responsive hydrogels have potential applications in multiple fields; however, a common problem is the brittleness of the resulting structures. Here, the authors report highly stretchable thermosensitive hydrogels by introducing sliding polyrotaxane cross-linkers and ionic groups into the system.

## Graphical abstract

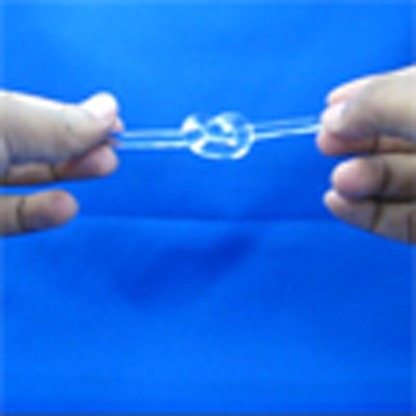

Numerous fundamental studies have investigated the chemistry and physics of polymer gels^1^. The properties of polymer gels, such as their molecular sieving ability, solvent retention and ability to serve as a corrective artificial lens, are already widely exploited in the pharmaceutical, medical and engineering fields. These polymer gels have broad utility in various fields, because the preparation of general polymer gels is exceedingly simple. For example, a poly(acrylamide) gel, which is used in electrophoresis in molecular biology, is usually prepared as follows: first, a predetermined quantity of monomer (acrylamide), cross-linker (*N*,*N*′-methylenebisacrylamide (BIS)) and initiator (for example, ammonium persulphate (APS)) are dissolved in pure water or a buffer solution. Next, the solution is deaerated under reduced pressure. Finally, the polymer gel is prepared by adding a few drops of accelerator (*N*,*N*,*N*′,*N*′-tetramethylethylenediamine (TEMED)) into the solution.

Currently, the study of stimuli-sensitive polymer gels, which undergo volume and shape changes in response to a stimulus, is becoming a highly active field of polymer gel research^2^,^3^. Stimuli-sensitive polymer gels that exhibit reversible changes in their selective permeability to molecules, surface wettability and ability to convert chemical energy into mechanical energy have been reported to be promising for drug delivery systems^4^, artificial muscles^5^,^6^ and sensors^7^,^8^,^9^,^10^. Although stimuli-sensitive polymer gels were first studied more than a few decades ago, they have not yet been commercialized for applications. One of the most significant problems is that conventional stimuli-sensitive polymer gels are usually mechanically weak or brittle. For example, chemically cross-linked stimuli-sensitive polymer gels are broken by only 1.2–1.5 times under uniaxial stretching^11^. In general, chemically cross-linked polymer gels (hereafter referred to as ‘chemical polymer gels’) exhibit rubber-like elasticity but are brittle and easily broken under deformation. The weak mechanical properties of chemical polymer gels are due to the inhomogeneous network structure generated during the polymerization, which are demonstrated by, for example, spatially non-uniform cross-linking densities and randomly distributed chain lengths between the cross-links^12^. When the shape of a bulk chemical polymer gel is distorted by an external force, the stress on the polymer network is concentrated on shorter polymer chains. As a result, the polymer network is mechanically disrupted. Similarly, when an abrupt environmental change leads to a modification in the volume or shape of a stimuli-sensitive chemical polymer gel, the polymer network fractures for the same reason^13^. Therefore, the weak mechanical properties of conventional stimuli-sensitive polymer gels make them unsuitable for practical applications.

Numerous attempts to improve the mechanical properties of polymer gels have been reported thus far^14^,^15^,^16^,^17^; most of the improved polymer gels have specially structured polymer networks, such as interpenetrating polymer networks^14^ and coupled networks between two types of four-armed polymers^16^, which enable them to exhibit excellent mechanical performance. However, the stimuli sensitivities of these chemical polymer gels have not been adequately investigated. In most cases, to obtain the desired stimuli-sensitive behaviour, the chemical components of these polymer gel networks must be altered by introducing stimuli-sensitive functional groups or monomers. Hydrogels that contain inorganic nanoparticles as cross-linkers can be easily prepared using stimuli-sensitive polymers and can be highly stretchable and tough^18^. However, the types of polymers that can be used to prepare these hydrogels are limited, and the hydrogels might dissolve in water during long-term utilization because the main cross-linking structures are hydrogen bonds between the inorganic nanoparticles and polymers.

To make stimuli-sensitive hydrogels for various practical applications, the mechanical properties of the existing well-studied stimuli-sensitive hydrogels should be improved retaining their stimuli sensitivities and using simple preparation techniques. This advance in stimuli-sensitive hydrogel research requires, for instance, a breakthrough in the cross-linking structure in conventional stimuli-sensitive hydrogels, although the cross-linking structure constitutes a small fraction of the hydrogels. On the basis of this requirement, a slide-ring gel developed by Okumura and Ito^19^ inspired us to prepare a new cross-linker for mechanically improved stimuli-sensitive hydrogels. This slide-ring gel is composed of polyrotaxane (PR)^20^, which consists of α-cyclodextrin (α-CD), polyethylene glycol (PEG) with terminal carboxylic acids and a capping agent (1-adamantanamine). In the slide-ring gel, α-CDs in one PR are cross-linked to α-CDs in different PRs. The PEG main chains are not fixed at the cross-linking points in the polymer network; instead, they can pass through the hole of a figure-8-shaped junction of cross-linked α-CDs freely, which is called the ‘pulley effect’. The concentration of stress on part of the polymer network is minimized through this effect. As a result, the slide-ring gel exhibits high extensibility and a small hysteresis on repeated extension and contraction.

Here we prepare thermosensitive hydrogels using PR as a cross-linker to exploit the pulley effect. *N*-Isopropylacrylamide (NIPA) is used as the monomer because it is known to form thermosensitive polymers and polymer gels in water^21^,^22^. We also introduce ionic sites into the PR-cross-linked polymer network using two different methods to obtain extremely stretchable and tough thermosensitive hydrogels. The ionic groups help the PR cross-linkers to become well extended in the polymer network. The resulting hydrogels are stretchable and tough, similar to soft rubbers, because the cross-linked α-CD molecules can move along the PEG chains.

## Results

### Past approach of hydrogels by PRs as cross-linkers

**Figure 1:**
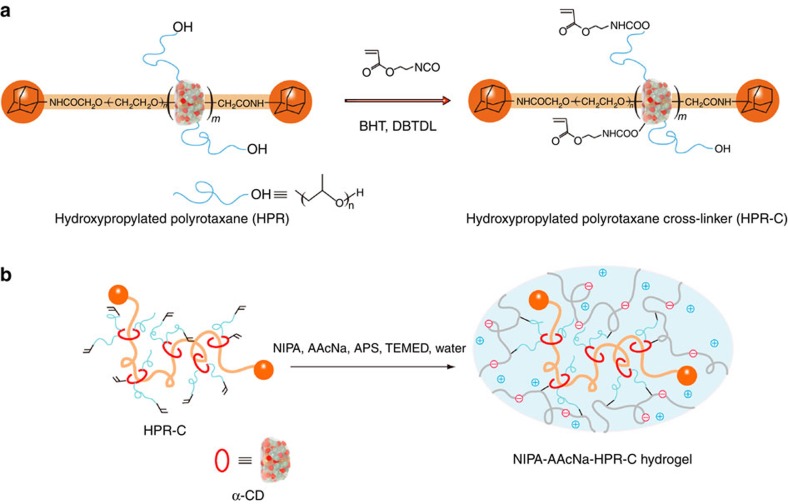
Preparation of the polyelectrolyte hydrogels using nonionic PR cross-linker. (**a**) Preparation of HPR-C from HPR, 2-acryloyloxyethyl isocyanate, DBTDL (catalyst) and BHT (polymerization inhibitor) in DMSO. (**b**) Preparation of the NIPA–AAcNa–HPR-C hydrogel from HPR-C (cross-linker), NIPA (main monomer), AAcNa (comonomer), APS (initiator) and TEMED (accelerator) in water.

Several previous attempts have been made to prepare stretchable hydrogels using PRs as the cross-linker. We first prepared a NIPA-based hydrogel using a PR modified by 2-acryloyloxyethyl isocyanate, which contains both isocyanate and vinyl groups, as the cross-linker (PR-C)^23^. The isocyanate groups form stable carbamate bonds with the α-CD hydroxyl groups in the PR to generate the cross-linking structures. It was expected that the NIPA–PR-C hydrogel would exhibit high extensibility, similar to the slide-ring gel composed of only the PR; however, the mechanical properties of the hydrogel were only slightly improved compared with those of chemical poly(NIPA) hydrogels prepared using conventional cross-linkers such as BIS. Although the NIPA–PR-C gel prepared in dimethyl sulphoxide (DMSO) was transparent, the gel became slightly opaque after replacing DMSO with water, even at 20 °C. These characteristics were attributed to the possibility that PR-C was in a contracted state in the hydrogel. The unmodified PR, which consists of α-CDs and PEG, is soluble only in a few solvents, such as DMSO, some ionic liquids, mixtures of organic amides and lithium salts and high-pH aqueous solutions. It is insoluble in pure water because the α-CD molecules aggregate due to hydrogen bonding between the hydroxyl groups on α-CDs^24^. Thus, α-CDs could not move along the PEG chains, and the pulley effect was restricted in water. As a result, the PR slide-ring gel appeared opaque and did not exhibit high extensibility. In high-pH aqueous solutions, such as a sodium hydroxide (NaOH) aqueous solution, the α-CDs do not aggregate through hydrogen bonding due to the ionization of the α-CD hydroxyl groups by NaOH; as a result, the slide-ring gel could swell^19^. However, a stimuli-sensitive hydrogel that is only useful in high-pH aqueous solutions does not have broad utility. Even when a nonionic, water-soluble hydroxypropylated PR cross-linker (HPR-C; Fig. 1a) was used, the resulting NIPA-based hydrogel did not exhibit greater extensibility than chemical poly(NIPA) hydrogels^25^. Although this hydrogel was nearly transparent, the pulley effect might not have worked well in this hydrogel because the solubility of HPR-C in water might be insufficient.

### Polyelectrolyte hydrogels by nonionic PR cross-linker

**Figure 2:**
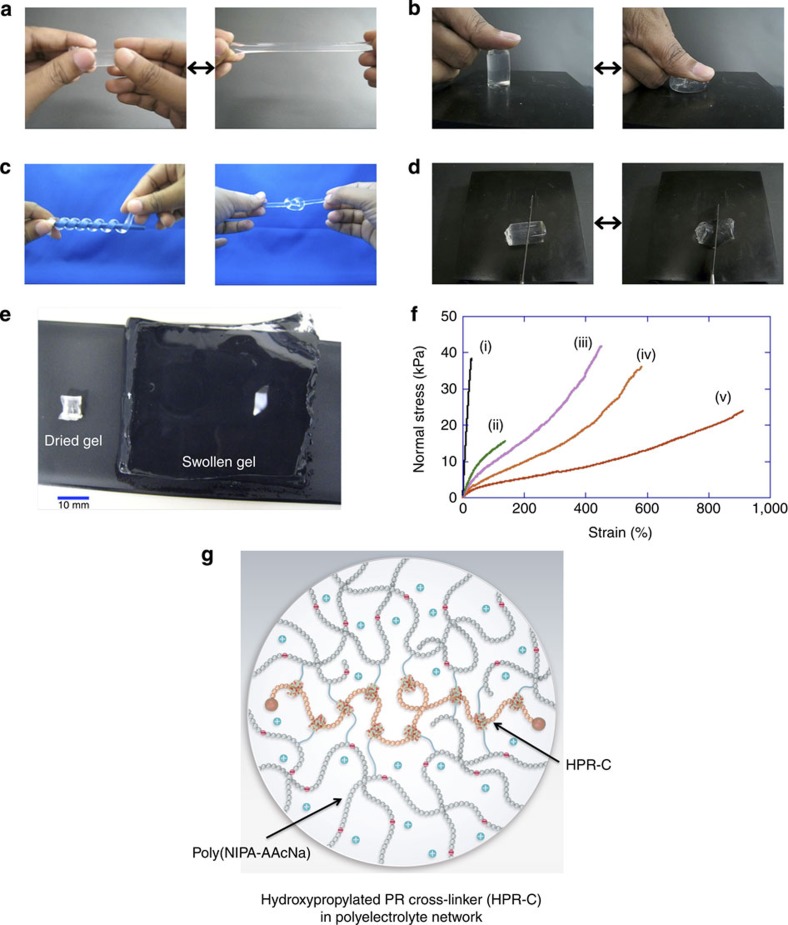
Properties of the polyelectrolyte hydrogels using nonionic PR cross-linker. (**a**) Elongated state of the NIPA–AAcNa–HPR-C hydrogel. (**b**) Compressed state of the NIPA–AAcNa–HPR-C hydrogel. (**c**) Coiled and knotted states of the NIPA–AAcNa–HPR-C hydrogel. (**d**) The NIPA–AAcNa–HPR-C hydrogel could not be easily cut with a knife. (**e**) Swelling of the NIPA–AAcNa–HPR-C hydrogel in water. Left: the dry gel (129 mg); right: the water-swollen gel (80 g). The NIPA–AAcNa–HPR-C hydrogel with 0.65 wt% of HPR-C absorbs up to ca. 62,000 wt% of water in its dry state. (**f**) Stress–strain curves of hydrogels: (i) NIPA–AAcNa–BIS (0.65 wt%), (ii) NIPA–AAcNa–BIS (0.065 wt%), (iii) NIPA–AAcNa–HPR-C (2.00 wt%), (iv) NIPA–AAcNa–HPR-C (1.21 wt%) and (v) NIPA–AAcNa–HPR-C (0.65 wt%). (**g**) Schematic of swollen HPR-C in the NIPA–AAcNa–HPR-C hydrogel.

If polyelectrolyte hydrogels are prepared using NIPA, the ionic monomer sodium acrylic acid (AAcNa), and HPR-C (Fig. 1b), the obtained hydrogels (NIPA–AAcNa–HPR-C hydrogels) are highly stretchable, flexible and durable when compressed (Fig. 2). Thus, the mechanical properties of these hydrogels are completely different from those of all existing chemical poly(NIPA) hydrogels and chemical poly(NIPA–AAcNa) hydrogels (Supplementary Movies 1 and 2). These hydrogels are so strong that they cannot be easily cut with a knife (Fig. 2d). Interestingly, the NIPA–AAcNa–HPR-C hydrogels rapidly shrink isotropically without undergoing any deformation at the gel surface (Supplementary Fig. 4). Furthermore, these hydrogels can absorb up to several tens of thousands percentage weight of water compared with their dried state. For example, the weight of a hydrogel with 0.65 wt% of HPR-C increases by up to 620-fold when it is placed in water (Fig. 2e). Figure 2f shows the stress–strain curves of NIPA–AAcNa–HPR-C hydrogels with different amounts of HPR-C and chemical poly(NIPA–AAcNa) hydrogels with different amounts of BIS. Young’s moduli, maximum elongation ratios and tensile strengths are summarized in Supplementary Table 2. For hydrogels with similar cross-linker concentrations (0.63–0.65 wt%), the maximum elongation of the hydrogels with the HPR-C cross-linker (912%) is substantially greater than that of the hydrogels with the BIS cross-linker (29%); the extensibility and toughness of the NIPA–AAcNa–HPR-C hydrogels are improved greatly by replacing the BIS cross-linker with HPR-C. In addition, NIPA–AAcNa–BIS (0.063 wt%) and NIPA–AAcNa–HPR-C (0.65 wt%) hydrogels have similar cross-linking density due to the presence of equal number of active vinyl groups in the cross-linkers BIS and HPR-C used for gelation (Supplementary Table 1). However, NIPA–AAcNa–BIS (0.063 wt%) hydrogel exhibits high stiffness and very poor tensile strength compared with NIPA–AAcNa–HPR-C (0.65 wt%) hydrogel. When the amount of HPR-C increases to 1.21 and 2.00 wt%, Young’s modulus and tensile strength are enhanced. In the NIPA–AAcNa–HPR-C hydrogels, the AAcNa component is ionized. The Na^+^ counter ions cannot stay near the ionized poly(NIPA–AAc^−^) polymer chains and thus move long distances away from them, making them stretch, to achieve a large gain in entropy^22^. The aggregation of the α-CD molecules at the HPR-C cross-linker can be prevented by stretching the poly(NIPA–AAc^−^) chains (Fig. 2g). As a result, the α-CD molecules can move along the PR main chains, and the pulley effect might occur.

**Figure 3:**
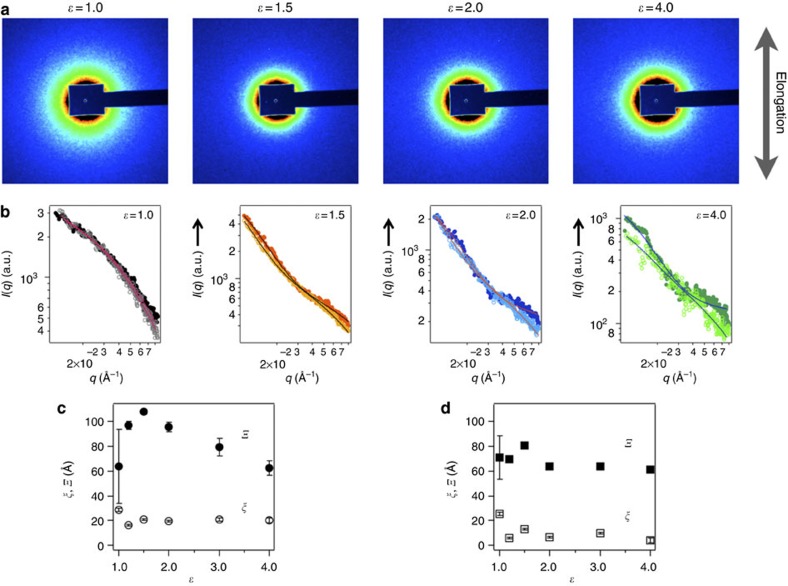
SAXS results of the polyelectrolyte hydrogels using nonionic PR cross-linker. (**a**) SAXS isointensity patterns of the NIPA–AAcNa–HPR-C hydrogel with 0.65 wt% of HPR-C for different elongations in the vertical direction. (**b**) Sector-averaged *I*(*q*) of the NIPA–AAcNa–HPR-C hydrogel with 0.65 wt% of HPR-C for different elongations in the parallel (open circles) and perpendicular (filled circles) directions. The solid lines are the equation (1) fitting results. (**c**) Stretching ratio dependence of *ξ* and Ξ for the NIPA–AAcNa–HPR-C hydrogel with 0.65 wt% of HPR-C in parallel to the elongation direction, and (**d**) stretching ratio dependence of *ξ* and Ξ for the NIPA–AAcNa–HPR-C hydrogel with 0.65 wt% of HPR-C in perpendicular to the elongation direction.

These excellent mechanical properties of this hydrogel are most likely achieved because of the homogeneous network structure afforded by the pulley effect. To confirm the structural homogeneity of the hydrogels, their structures were analysed under uniaxial elongation by small-angle X-ray scattering (SAXS). In general, the spatial inhomogeneity of cross-links is hidden by the fluctuation in the polymer chain concentration before the elongation. When the chemical gels are deformed, the inhomogeneous structure is exposed and the two-dimensional (2D) X-ray or neutron scattering patterns become elliptical^26^. Figure 3a shows 2D SAXS patterns of as-prepared (elongation ratio *ε*=1) and vertically stretched (*ε*>1) NIPA–AAcNa–HPR-C hydrogels cross-linked with 0.65 wt% HPR-C. These patterns are almost isotropic, which is consistent with the results for slide-ring gels in a good solvent^27^. This hydrogel contains water, which is a good solvent; therefore, it has no internal aggregation structure. The SAXS pattern of this hydrogel remains isotropic when the hydrogel is elongated by a factor of greater than four, which is in contrast to the elliptical pattern observed for a poly(acrylamide) gel when elongated by a factor of 1.5 (ref. 27)^27^. We quantitatively evaluated the SAXS patterns by calculating the sector average of the intensity in the directions of parallel and perpendicular to the elongation. The angle of the sector was 20° in each direction. Figure 3b shows the sector-averaged scattering profiles of the hydrogel at elongation ratios of *ε*=1–4 in each direction. The scattering function for polymer gels in a good solvent is given as follows^28^,^29^:


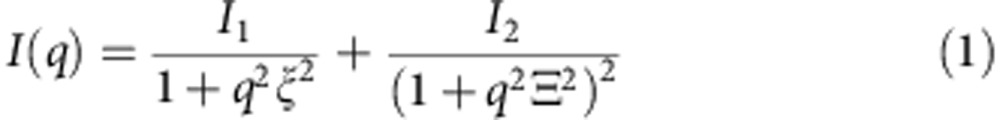


The first term on the right side of equation (1) is a Lorentzian function (that is, an Ornstein–Zernike function) that describes the polymer concentration fluctuation, and the second term is a squared Lorentzian function (that is, a Debye–Bueche function) that describes excess scattering from a spatial inhomogeneity. The parameter *q* is the magnitude of the scattering vector, *ξ* is the correlation length of the fluctuation and Ξ is the characteristic length of the inhomogeneity. The *I*_1_ and *I*_2_ terms are the scattering intensities at *q*=0 that correspond to the static and dynamic contributions, respectively. An analysis of the sector-averaged intensity profile using equation (1) gives *ξ* and Ξ as fitting parameters. According to the results of previous SAXS studies on conventional chemical gels, Ξ in the elongation direction increases as the elongation ratio increases, which indicates that conventional chemical gels have spatial inhomogeneities in their cross-linking structures that are magnified on elongation^30^. The fitting results are shown in the scattering profiles in Fig. 3b. Figure 3c,d shows the elongation ratio dependence of *ξ* and Ξ in each direction. The *ξ* parameter is nearly constant at ~10–20 Å for all the elongation ratios studied. For elongated gels, the Ξ parameter does not change significantly or slightly decreases as the elongation ratio increases, which indicates that the inhomogeneity of this hydrogel is much lower than that of the conventional chemical hydrogels. This behaviour is the same as that observed in small-angle neutron scattering studies of slide-ring gels^31^ and is significantly different from that of chemical gels^30^. Thus, the SAXS results in this study demonstrate the spatial homogeneity of the polymer network and the pulley effect in the NIPA–AAcNa–HPR-C hydrogels.

### Hydrogels using ionic PR cross-linker

Furthermore, to obtain a wide variety of thermosensitive hydrogels with mechanical toughness and superb functionality, the thermo-responsiveness must be efficiently regulated while maintaining the enhanced mechanical properties. Thus, we demonstrate a case in which the thermo- and pH-responses of PR-cross-linked hydrogels are regulated by controlling the spatial distribution of the ion concentration in the hydrogels.

The pure poly(NIPA) hydrogel without any ionic species undergoes a volume change at 32 °C (ref. 21). For hydrogels consisting of randomly copolymerized NIPA and ionic monomers, the characteristic temperature of the volume change generally increases with increasing ionic monomer concentration. However, the volume of the hydrogel does not change when a large amount of ionic monomers are present in the hydrogel^22^; the strongly hydrated ions distributed throughout the whole polymer network inhibit the dehydration of the polymer chains. Interestingly, the temperature-sensitive swelling behaviours of random and graft copolymer hydrogels composed of NIPA and ionic monomers are dramatically different. The volume-change temperatures of the graft copolymer hydrogels have been reported to be similar to that of the pure poly(NIPA) hydrogel because the coil-globule transition of the poly(NIPA) portion is not affected by the grafted polymer composed of ionic monomers^32^,^33^.

**Figure 4:**
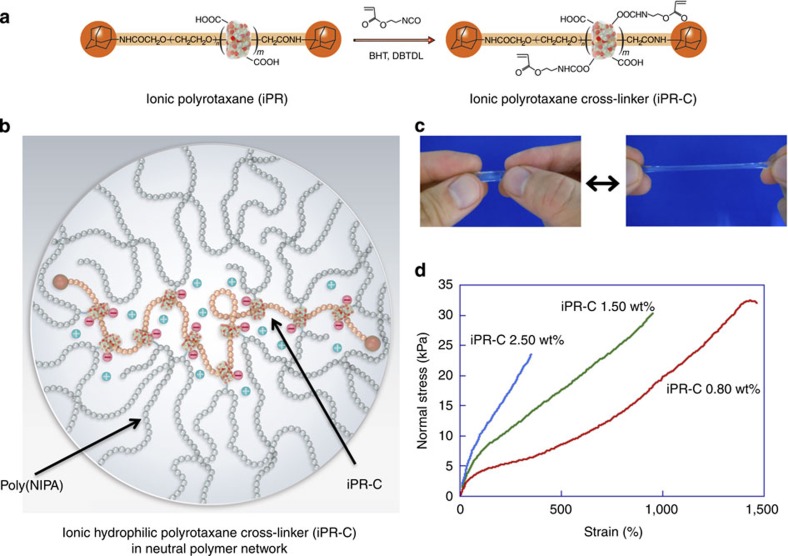
Preparation of the hydrogels using ionic PR cross-linker. (**a**) Preparation of iPR-C from iPR, 2-acryloyloxyethyl isocyanate, DBTDL (catalyst) and BHT (polymerization inhibitor) in DMSO. (**b**) Schematic of swollen iPR-C in the NIPA–iPR-C hydrogel. (**c**) Elongated state of the NIPA–iPR-C hydrogel. (**d**) Stress–strain curves for NIPA–iPR-C hydrogels with different amounts of iPR-C.

**Figure 5:**
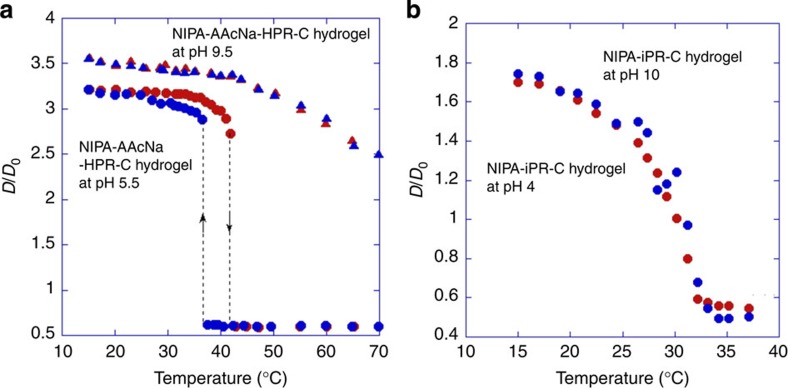
Swelling behaviours of the polyelectrolyte hydrogels using nonionic PR cross-linker and the hydrogels using ionic PR cross-linker. The degree of swelling *D*/*D*_0_ for the (**a**) NIPA–AAcNa–HPR-C hydrogel with 0.65 wt% of HPR-C during both heating (red) and cooling (blue) processes, and (**b**) NIPA–iPR-C hydrogel with 0.80 wt% of iPR-C during heating process in aqueous solutions with different pHs as a function of temperature. *D* and *D*_0_ denote the gel diameters at equilibrium and on synthesis, respectively.

On the basis of the graft copolymer hydrogel properties, we successfully prepared pure poly(NIPA) hydrogels without ions that exhibit both mechanical toughness and thermo-responsiveness utilizing an ionic PR derivative as a cross-linker (Fig. 4a). This PR cross-linker (iPR-C) has carboxyl groups on the α-CD molecules, making it highly water soluble. It was expected that iPR-C can be well extended in a neutral poly(NIPA) network (Fig. 4b), as observed for HPR-C in the polyelectrolyte network (Fig. 2g). The most distinctive feature of the iPR-C gels is that the ionic groups are localized only on the PR cross-linker. The spatial distribution of the ionic groups on the polymer network is therefore remarkably different from that in the randomly copolymerized NIPA–AAcNa gels. As expected, the iPR-C hydrogels are as transparent, stretchable and tough in pure water (Fig. 4d; Supplementary Table 3; Supplementary Movie 3) as the NIPA–AAcNa–HPR-C hydrogels. The degree of dissociation of the carboxylic acid groups in the NIPA–AAcNa–HPR-C hydrogels changes with pH, and the osmotic pressure in the hydrogels therefore changes with pH because the ionic AAcNa monomer is distributed throughout the polymer network. As a result, the swelling behaviour of the NIPA–AAcNa–HPR-C hydrogels is highly dependent on the solvent pH (Fig. 5a). However, the swelling behaviour of the NIPA–iPR-C hydrogels shows little dependence on the solvent pH when the amount of iPR-C is lower than 3 wt% (Supplementary Figs 5 and 6). Although the carboxyl groups of iPR-C can also dissociate in water in response to changes in the pH, the dissociated ion does not affect the transition temperature significantly because the amount of iPR-C is very small and the ionic groups are localized only on the iPR-C cross-linker. Thus, the pH dependence of the temperature response is successfully regulated by controlling the spatial distribution of the ionic groups in the hydrogels via simple chemical modifications of the PR.

## Discussion

We demonstrate the simple preparation of extremely stretchable hydrogels with temperature and pH sensitivities by using PR derivatives as cross-linkers and introducing ionic sites into the polymer network via two different methods. One method involves randomly copolymerizing NIPA and ionic AAcNa with HPR-C as the cross-linker. Because of the considerable swelling power of the polyelectrolyte hydrogels, HPR-C, which might be in a contracted state in the poly(NIPA) hydrogel, achieves a swollen coil state, and the pulley effect is observed. The resulting hydrogels exhibit significant pH sensitivity because of the electrostatic repulsion between the charges on the polymer chains and the effect of pH on the osmotic pressure of the counter ions. This finding is significant for improving existing polyelectrolyte hydrogels. Although current polyelectrolyte hydrogels have the potential to absorb and release large amounts of solvents^34^, recognize or react with specific molecules^35^,^36^,^37^ and be employed in artificial muscles^5^,^6^, their brittleness must be reduced before they can be used in these applications. The PR-based cross-linker developed here can lead to significant improvements in the mechanical properties of existing polyelectrolyte hydrogels without compromising their characteristic properties. Alternatively, iPR-C can be widely employed as a cross-linker to improve the mechanical properties of a broad range of existing polymer gels, including non-polyelectrolyte gels, because it does not require the use of an ionic monomer to obtain enhanced mechanical properties. Therefore, the mechanical properties of existing well-studied stimuli-sensitive hydrogels can be improved without compromising their stimuli sensitivities by using iPR-C.

In conclusion, extremely stretchable and tough thermosensitive hydrogels were obtained by using PR cross-linkers and introducing ionic sites into the polymer network. Two different preparation methods were employed. Our results indicate that using a small amount of a supramolecular building block as a cross-linker in a polymer network results in dramatic changes in its mechanical properties. Using the preparation methods described here, which are simple, like conventional polymer gel preparation techniques, the mechanical properties of various polymer gels can be improved. Thus, stimuli-sensitive hydrogels with good mechanical performance and controllable functionality can be produced for drug delivery systems, tissue engineering, artificial muscles and sensors. Substantial progress is being made towards the practical use of stimuli-sensitive hydrogels for these applications.

## Methods

### Materials

HPR (*M*_n_=126,000; *M*_w_/*M*_n_=1.4; inclusion ratio of α-CD: 29%) and ionic PR (iPR: *M*_n_=156,000; *M*_w_/*M*_n_=1.4; inclusion ratio of α-CD: 23%) were purchased from Advanced Soft Materials (Kashiwa, Japan) and were used without further purification. NIPA was purified by recrystallization from toluene/*n*-hexane. AAcNa (Sigma-Aldrich, MO, USA), 2,2′-methylenebisacrylamide (BIS; Acros Organics, Geel, Belgium), APS (Acros Organics), TEMED (Wako Pure Chemical Industries, Tokyo, Japan), dibutyltin dilaurate (DBTDL; Tokyo Kasei Kogyo, Tokyo, Japan) and butyl hydroxyl toluene (BHT; Tokyo Kasei Kogyo) were purchased as reagent-grade materials and used as received. 2-acryloyloxyethyl isocyanate (Showa Denko K.K., Japan) was used as received. Milli-Q ultra-pure water was used in all the experiments. 

### Preparation of PR cross-linkers (HPR-C and iPR-C)

HPR or iPR (500 mg), the DBTDL catalyst (1 drop) and BHT (polymerization inhibitor, 0.78 mg) were dissolved in 30 ml of anhydrous DMSO. 2-Acryloyloxyethyl isocyanate (78 mg) was dissolved in 10 ml of anhydrous DMSO and the solution was added dropwise to the mixtures with vigorous stirring in the absence of light. The mixtures were then continuously stirred overnight at 40 °C to ensure that the reactions were complete. HPR-C or iPR-C was reprecipitated from the reaction mixture using an excess of methanol or acetone, respectively, and the precipitated product was refrigerated. The products were washed several times with methanol and acetone and then dried. The total number of vinyl groups per HPR and iPR was estimated from ^1^H-NMR spectra and was found to be *ca.* 200 (Supplementary Figs 1–3).

### Preparation of hydrogels

The stretchable hydrogels were prepared by conventional free-radical polymerization of the monomers with the PR cross-linkers. For the polyelectrolyte hydrogels, appropriate amounts of NIPA, AAcNa, HPR-C or BIS, and APS (initiator) were dissolved in water. The final concentrations of the ionic monomer and NIPA were 0.1 and 1.9 M, respectively, whereas the cross-linker concentration changed. For the NIPA–iPR-C hydrogel, appropriate amounts of NIPA, iPR-C and APS were dissolved in water. The final concentration of NIPA monomer was 2 M, whereas the concentration of iPR-C changed. In all the pre-gel solutions, the total concentration of the monomers excluding the cross-linkers was fixed at 2 M. Next, N_2_ gas was bubbled through the pre-gel solutions for 30 min, which were then sonicated to remove excess nitrogen from the solution. The pre-gel solution was infused onto glass slides separated by Teflon spacers to prepare slab gels. Cylindrical gels were prepared using microcapillaries with an inner diameter of 270 μm. To initiate the polymerization below room temperature, a few drops of TEMED were added to the pre-gel solution. The gelations were performed at 4 °C for 24 h. The hydrogels were thoroughly washed with water for 2 weeks to remove any unreacted monomer or reaction residue.

### Mechanical strength tests

The mechanical properties of the hydrogels were measured with a TA Instruments RSA III rheometric solid analyser. The as-prepared 0.5-mm-thick hydrogel samples were cut into 5-mm × 30-mm rectangles for the stress–strain measurements. The hydrogel samples were placed and aligned between two fixtures and were tightly fixed by sample-loading bolt screws at both ends. We ensured that the hydrogel samples were smooth before each measurement. The transient strain-controlled multiple extension mode was used as the test set-up. The elongation rate was 0.1 mm s^−1^. The measurements were conducted in air at room temperature (~25 °C). The reproducibility of the stress–strain curves was verified by successive measurements.

### SAXS

SAXS measurements were performed using the synchrotron radiation light source (beamlines BL-6A, BL-15A and BL-10C) at the Photon Factory, High Energy Accelerator Research Organization, KEK (Tsukuba, Japan). The wavelength *λ* of the X-ray beam was 1.50 Å, and the beam size was 0.7 mm (vertical) × 0.7 mm (horizontal). An X-ray charge-coupled device detector (C4880-50-26, Hamamatsu Photonics, Japan) equipped with a 150-mm diameter X-ray image intensifier was used as the SAXS detector. The as-prepared 1-mm-thick hydrogel samples were cut into rectangles before they were analysed. The samples were placed in a uniaxial stretching machine for the deformation. The stretching direction of the hydrogel samples was vertical. The distance between the sample and the detector was ~2,300 mm, which was later calibrated according to the diffraction peaks of chicken tendon collagen (domain spacing, *d*=653 Å). The SAXS patterns were recorded for 0.01 Å^−1^<*q*<0.08 Å^−1^, where *q* is the magnitude of the scattering vector and 2*q* is the scattering angle. The typical exposure time was 30 s, where the scattering pattern did not change. Therefore, the beam damage and the drying of the gel were ignorable in this experimental condition.

## Author contributions

Y.T. designed the project. A.B.I., K.E., H.G. and Y.S. performed the experiments. All authors discussed the results and contributed to the data interpretation. Y.T., Y.S. and A.B.I. wrote the manuscript. Y.T., Y.S., A.B.I., K.I. and T.S. commented on the manuscript.

## Additional information

**How to cite this article:** Bin Imran, A. *et al.* Extremely stretchable thermosensitive hydrogels by introducing slide-ring polyrotaxane cross-linkers and ionic groups into the polymer network. *Nat. Commun.* 5:5124 doi: 10.1038/ncomms6124 (2014).

## Supplementary Material

Supplementary Figures and Supplementary Tables Supplementary Figures 1-6 and Supplementary Tables 1-3

Supplementary Movie 1 Mechanical Properties of NIPA-AAcNa-BIS hydrogel

Supplementary Movie 2 Mechanical Properties of NIPA-AAcNa-HPR-C hydrogel

Supplementary Movie 3 Mechanical Properties of NIPA-iPR-C hydrogel
