# Supplementary material for: Extremely stretchable thermosensitive hydrogels by introducing slide-ring polyrotaxane cross-linkers and ionic groups into the polymer network
Source: Nat Commun. 2014 Oct 8;5:5124. doi: 10.1038/ncomms6124 (PMC4214411; doi:10.1038/ncomms6124)
Supplement: Supplementary Figures and Supplementary Tables — Supplementary Figures 1-6 and Supplementary Tables 1-3 [file ncomms6124-s1.pdf]

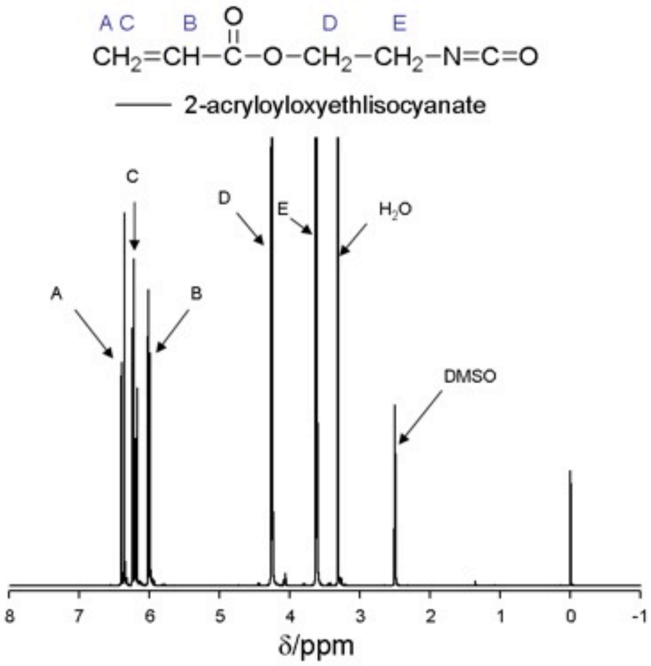

Supplementary Figure 1. ^{1}H-NMR spectrum of 2-acryloyloxyethyl isocyanate in DMSO-d_6.

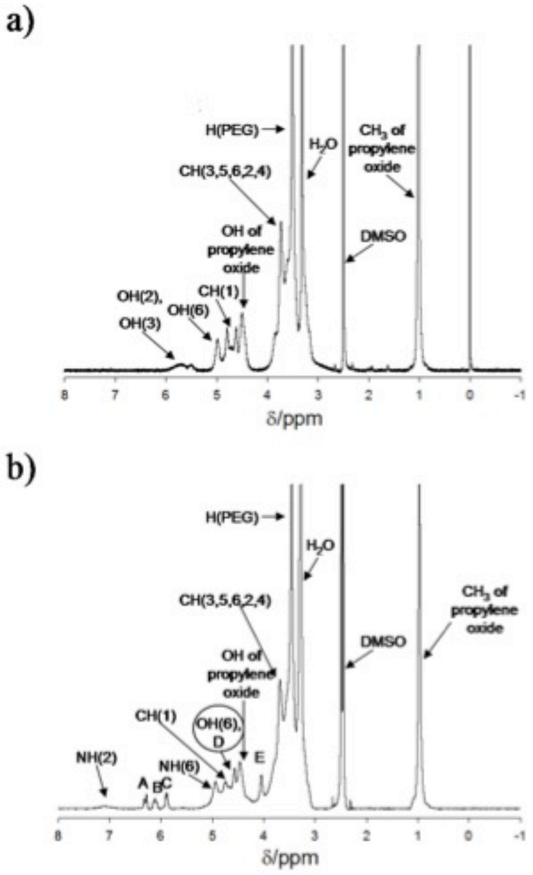

Supplementary Figure 2. ^{1}H-NMR spectra of a) HPR and b) HPR-C in DMSO-d_6. All the characteristic peaks of HPR and HPR-C arise from α-CD and the modified α-CD group (the number in parentheses indicates the carbon position of the 1,4-linked α-D-glucopyranoside unit of α-CD). The number of vinyl groups per α-CD unit of HPR-C is 1.13.

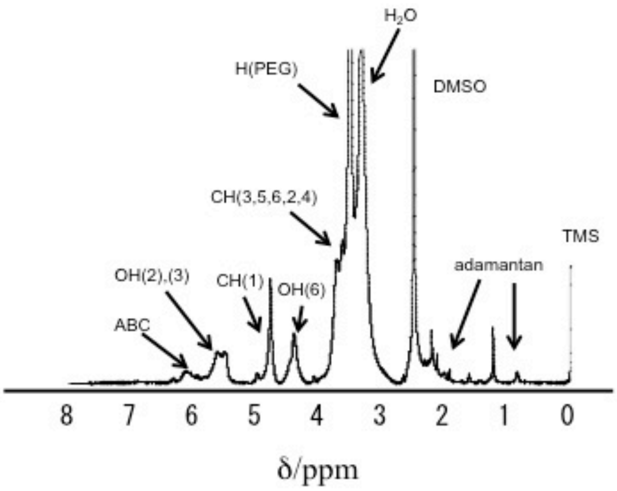

Supplementary Figure 3. ^{1}H-NMR spectrum of iPR-C in DMSO-d_6. The number of vinyl groups per α-CD unit of iPR-C is 1.30.

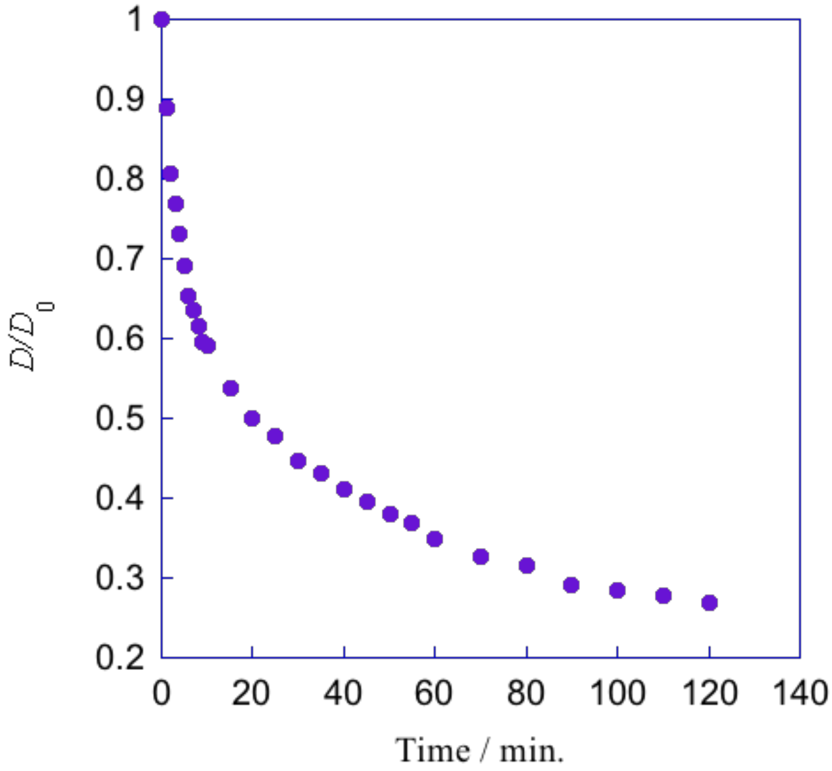

Supplementary Figure 4. Shrinking kinetics of the NIPA–AAcNa–HPR-C hydrogel with 0.65 wt% of HPR-C after a temperature jump from 20 to 50°C. The cylindrical gels were kept in the desired solvent in a glass cell. Two circulators fixed at two different target temperatures were connected to the glass cell. By changing the solvent path, the temperature inside the cell could be quickly changed from one temperature to the other. The temperature inside the cell was stable to ± 0.2°C for a few hours. The target temperature was reached approximately 70 ± 5 sec after switching. Videos of the morphological changes of the investigated gels were recorded using BUFFALO PCast TV capture software on a computer. Pictures were captured from the videos at different time intervals.

The NIPA-AAcNa-HPR-C hydrogels rapidly shrink isotropically without undergoing any deformation at the gel surface. When HPR-C is used for gelation, the resulting gel not only possesses a cross-linked polymer network but also free, dangling polymer chains attached to the α-CD active groups of the cross-linkers. The presence of dangling polymer chains with one free end might aid in a smooth rearrangement to the final shrunken state, explaining the absence of deformations at the gel surface when the NIPA-AAcNa-HPR-C hydrogels quickly shrink to an equilibrium state. Further investigations will provide more details about this process.

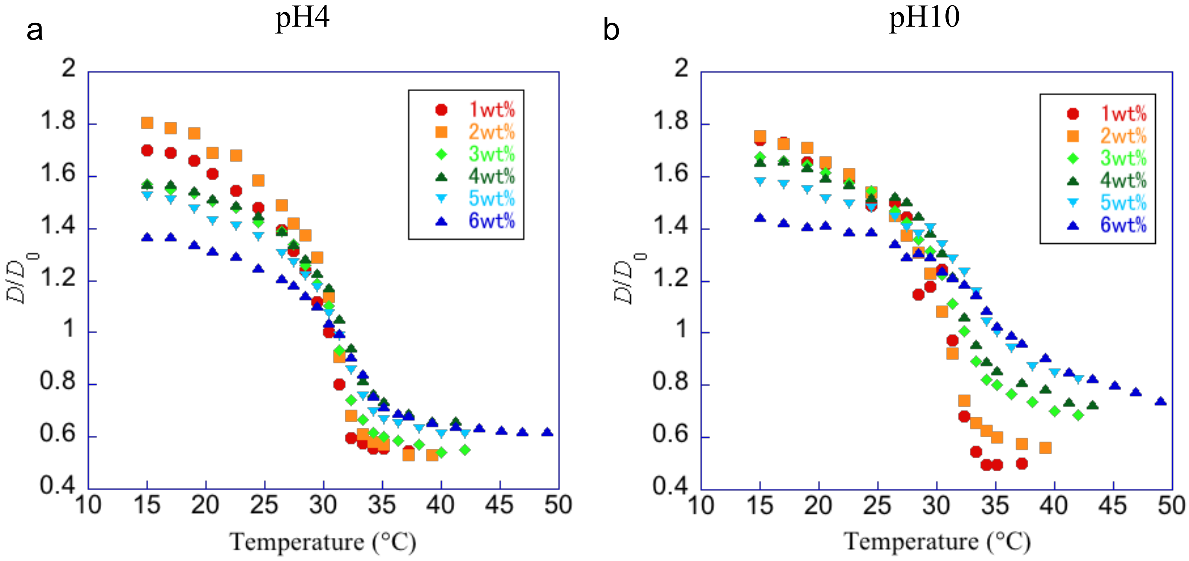

Supplementary Figure 5. Temperature dependence of the degree of swelling D/D_0 for NIPA-iPR-C hydrogels containing different amounts of iPR-C in an aqueous buffer solution at a) pH 4 and b) pH 10.

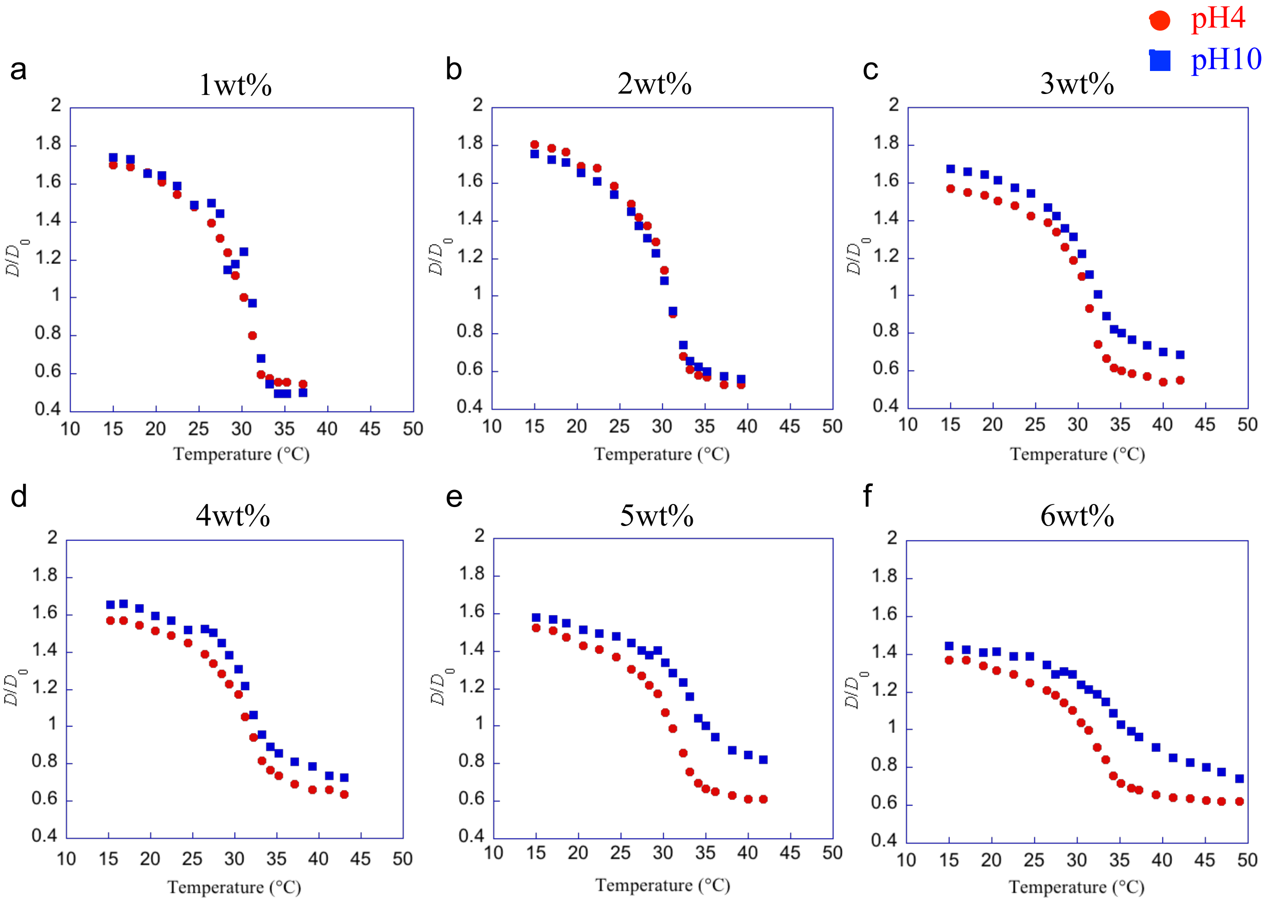

Supplementary Figure 6. Temperature dependence of the degree of swelling D/D_0 for NIPA-iPR-C hydrogels containing a) 1 wt%, b) 2 wt%, c) 3 wt%, d) 4 wt%, e) 5 wt%, and f) 6 wt% of iPR-C in different aqueous buffer solutions. These results are reconstructed from Supplementary Figure 5.

Supplementary Table 1. Preparation condition of NIPA–AAcNa-BIS and NIPA-AAcNa-HPR-C hydrogels (in Figure 2f) by varying the amounts of cross-linkers BIS and HPR-C. *h* and *n* denote the water content in as-prepared gel and number of active vinyl groups, respectively.

Hydrogels code	NIPA (g)	AACNa (g)	BIS (mg)	HPR-C (mg)	TEMED (μL)	APS (mg)	water (g)	*h* (%)	*n*
NIPA-AAcNa-BIS (0.63 wt%)	1.075	0.047	38.5	0	20	6	5.00	78.5	3.0×10^{20}
NIPA-AAcNa-BIS (0.063 wt%)	1.075	0.047	3.85	0	20	6	5.00	78.9	3.0×10^{19}
NIPA-AAcNa-HPR-C (2.02 wt%)	1.075	0.047	0	125	20	6	5.00	77.5	9.3×10^{19}
NIPA-AAcNa-HPR-C (1.21 wt%)	1.075	0.047	0	75	20	6	5.00	78.0	5.6×10^{19}
NIPA-AAcNa-HPR-C (0.65 wt%)	1.075	0.047	0	40	20	6	5.00	78.5	3.0×10^{19}

Supplementary Table 2. Young's moduli, maximum elongation ratios, and tensile strengths of NIPA–AAcNa hydrogels cross-linked by either BIS or HPR-C.

cross-linker	cross-linker content [wt%]	Young's modulus [kPa]	maximum elongation [%]	tensile strength [kPa]
BIS	0.063	15.5	138	15.5
	0.63	37.8	29	38.3
HPR-C	0.65	23.9	912	23.9
	1.21	36.1	583	36.6
	2.00	43.2	451	40.9

Supplementary Table 3. Young's moduli, maximum elongation ratios, and tensile strengths of NIPA hydrogels cross-linked by iPR-C.

cross-linker	cross-linker content [wt%]	Young's modulus [kPa]	maximum elongation [%]	tensile strength [kPa]
iPR-C	0.80	8.32	1463	31.4
	1.50	10.1	953	29.3
	2.50	14.2	354	23.4
